# Learning Background-Suppressed Dual-Regression Correlation Filters for Visual Tracking

**DOI:** 10.3390/s23135972

**Published:** 2023-06-27

**Authors:** Jianzhong He, Yuanfa Ji, Xiyan Sun, Sunyong Wu, Chunping Wu, Yuxiang Chen

**Affiliations:** 1 School of Information and Communication, Guilin University of Electronic Technology, Guilin 541004, China; hjz17805972248@163.com (J.H.); wuchunping1@163.com (C.W.); 18859135897@163.com (Y.C.); 2 National & Local Joint Engineering Research Center of Satellite Navigation Positioning and Location Service, Guilin University of Electronic Technology, Guilin 541004, China; 3 Guangxi Key Laboratory of Precision Navigation Technology and Application, Guilin University of Electronic Technology, Guilin 541004, China; 4 GUET-Nanning E-Tech Research Institute Co., Ltd., Nanning 530031, China; 5 School of Mathematical and Computational Sciences, Guilin University of Electronic Technology, Guilin 541004, China; wusunyong121991@163.com

**Keywords:** visual object tracking, discriminative correlation filter, background suppressed, dual regression, response aberration

## Abstract

The discriminative correlation filter (DCF)-based tracking method has shown good accuracy and efficiency in visual tracking. However, the periodic assumption of sample space causes unwanted boundary effects, restricting the tracker’s ability to distinguish between the target and background. Additionally, in the real tracking environment, interference factors such as occlusion, background clutter, and illumination changes cause response aberration and, thus, tracking failure. To address these issues, this work proposed a novel tracking method named the background-suppressed dual-regression correlation filter (BSDCF) for visual tracking. First, we utilize the background-suppressed function to crop out the target features from the global features. In the training step, while introducing the spatial regularity constraint and background response suppression regularization, we construct a dual regression structure to train the target and global filters separately. The aim is to exploit the difference between the output response maps for mutual constraint to highlight the target and suppress the background interference. Furthermore, in the detection step, the global response can be enhanced by a weighted fusion of the target response to further improve the tracking performance in complex scenes. Finally, extensive experiments are conducted on three public benchmarks (including OTB100, TC128, and UAVDT), and the experimental results indicate that the proposed BSDCF tracker achieves tracking performance comparable to many state-of-the-art (SOTA) trackers in a variety of complex situations.

## 1. Introduction

Visual object tracking aims to achieve stable and accurate target localization in subsequent video frames based on the limited information given in the first frame [1]. As one of the hot topics in the field of computer vision, it has been widely applicated in intelligent monitoring [2], robotics [3], traffic control [4], and uncrewed aerial vehicle (UAV) reconnaissance [5]. Nevertheless, visual object tracking tasks in real scenarios still suffer from numerous challenging problems, such as occlusion, illumination changes, background clutters, and deformation, often leading to tracking failure.

In recent years, discriminative correlation filter (DCF)-based tracking methods have received a great deal of attention due to their computational efficiency and sufficient tracking performance. With the circular sampling operation and fast Fourier transform (FFT) technology, DCF can easily obtain a significant quantity of training samples for tracker learning and convert the time-consuming correlation operation in the spatial domain to simple element-wise multiplication in the frequency domain, effectively reducing the computational complexity and increasing the tracking speed. 

However, the periodic assumption introduces unwanted boundary effects. Due to the cyclic shift operation, the background region is mostly replaced by duplicate image patches. The produced detection and training samples, except for the positive samples in the target’s surroundings, the negative samples in the background region are all non-real synthetic samples, which leads to a lack of learning of the real background information by the model and severely weakens its ability to distinguish the target from the background. For this, two representative improvement directions have emerged. One is introducing spatial constraints to penalize the filter coefficients in the boundary region [6,7,8]. The other is to improve the quality of training samples by imposing constraints on the spatial domain features [9,10,11]. Both are based on expanding the detection region to obtain as much realistic background information as possible, which leads to improved filter discriminative power. However, the lack of a strategy to cope with response aberration in complex tracking scenarios makes the performance of the above trackers sub-optimal.

The response aberration caused by background interference has a significant impact on the tracking results. DCF adopts ridge regression for filter training and uses it as a classifier to discriminate between the target and background [12]. The label of the regression term is a normally distributed two-dimensional matrix, which aims to obtain an output response map with an ideal normal distribution, i.e., a clear single peak in the target region and a flat one in the background region. The more prominent the peak of the response map, the flatter the response in the background region, and the more reliable the tracking results will be [13]. With a large amount of background interference, the object’s appearance tends to change dramatically, while the response map presents a multi-peak trend. In severe cases, the maximum peak position will no longer be the target position, resulting in tracking failure. To reduce the influence of response aberration, multiple high-confidence model update strategies [13,14,15,16] were proposed, which expect to construct feedback loops by analyzing the quality of response maps to guide the appearance model update. Thus, it can maintain a relatively pure training sample in complex scenarios, improving the tracker’s discriminative power. However, such passive learning strategies may not be effective, as they do not prevent the occurrence of response aberrations. To achieve background suppression, some methods [17,18,19,20] use the known previous frame’s response as a template to limit the variation rate of the current frame’s response, thus effectively limiting drastic response changes. Some trackers [6,17,21,22] suppress the background in a very straightforward way, i.e., using a spatial constraint matrix to mask or ignore the background region. However, this also inevitably involves error accumulation or lost partial view information, which limits the filter’s discriminative power.

**Figure 1:**
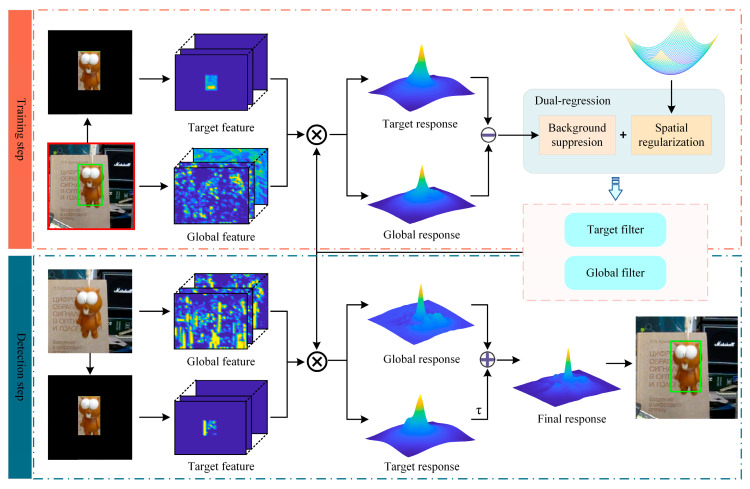
Overall framework of the proposed BSDCF tracker.

In this work, we propose a novel background-suppressed dual-regression correlation filter (BSDCF) for visual tracking, which utilizes the BACF [10] as the baseline tracker. The proposed BSDCF tracker’s overall framework is shown in Figure 1. Specifically, we employ the background suppressed function to crop the target features from the global features and construct a dual regression model to train the target filter and the global filter separately. In the training step, the difference between the output response maps for mutual constraint highlights the target and suppresses the response aberration. In the detection step, as the target filter learns sufficient target information, the global response can be enhanced by a weighted fusion of the target response to further improve the tracking performance in complex scenes. The main contributions can be summarized in the following:

- We proposed a novel background-suppressed dual-regression correlation filter (BSDCF) for visual tracking, which adopts the overall strategy of exploiting the target response to restrict the background response’s change rate for addressing the response aberration due to background interference;
- Using the alternating direction method of multipliers (ADMM) algorithm [23], the proposed BSDCF can efficiently solve the closed-form solutions;
- The overall experimental results at the OTB100, TC128, and UAVDT benchmarks show that the performance of the proposed BSDCF is competitive compared to other 13 state-of-the-art (SOTA) trackers, and it can perform better tracking performance in complex tracking scenarios.

## 2. Related Works

The discriminative correlation filters (DCF)-based tracking methods have succeeded greatly in recent years. In this section, we briefly review the related tracking approaches, which include the correlation filter tracking algorithms, mitigating boundary-effects-based tracking methods, and background suppressed-based trackers. 

### 2.1. Tacking with DCF

Bolme et al. [24] proposed the earliest DCF that converts the time-consuming matrix correlation operation in the spatial domain to element-wise multiplication in the frequency domain, effectively reducing the algorithm’s complexity and achieving an impressive tracking speed of 669 fps. Henriques et al. [12] introduced the cyclic matrix for dense sampling, which is a way to expand the number of training samples by shifting the original image itself. Meanwhile, they exploited kernel-trick to solve the nonlinear classification problem. Since then, the preliminary work of DCF has matured. Most of the subsequent researchers are devoted to the targeted improvement of the weaknesses of CSK/KCF, which mainly contains: enhancing the target expression [25,26,27,28,29,30,31,32], improving the scale adaptation ability [33,34,35,36,37], and model optimization [13,16,38,39,40,41], etc. 

An accurate and robust target representation is particularly important for tracker performance improvement. In practice, most early correlation filters used single-channel grayscale features, but its representation power for objects has difficulty supporting complex tracking tasks. To this end, scholars have tried to fuse different hand-crafted features [25,27,29,32,42] and deep features [26,28,31,43] into the correlation filter framework, significantly improving the overall tracking performance. Meanwhile, to solve the problem of fixed templates failing to reflect the object’s scale changes, Li et al. [36] proposed SAMF (scale adaptive kernel correlation filters), which scales the search area according to the pre-defined seven scale factors and then obtains the target location and optimal scale by retrieving the response maximum points in the detection phase. Danelljan et al. [37] proposed the discriminative scale space tracker (DSST), which adds an additional one-dimensional scale filter to the two-dimensional position filter, aiming to partition the target localization and scale estimation tasks. To increase the tracking speed, the fast version of the DSST (fDSST) [35] employs feature reduction and QR decomposition techniques to further reduce the computational complexity. To further improve the filter tracking performance, many researchers have improved the model structure or optimization methods, which have greatly promoted the development of DCF. Tang et al. [41] considered the upper bound of the multi-kernel correlation filter (MKCF) as its objective function and introduced historical views as training samples to train the filter. Wang et al. [38] incorporate the contextual information of the target as negative samples to enhance the model’s ability to perceive the background and use saliency features to augment the filter templates. 

### 2.2. DCF Tacking with Boundary Effects

The circular shift sampling significantly expands the number of training samples but also causes unwanted boundary effects. To overcome this limitation, the spatially regularized discriminative correlation filters (SRDCF) [6] penalize the filter coefficients in the boundary region by constructing spatial constraints, which enable it to learn the background information in a larger search domain without worrying about introducing much background interference. Additionally, by exploiting a binary mask matrix, the background-aware correlation filter (BACF) [10] makes it possible to crop the real background images in a large search domain, thus effectively improving the quality of the training samples. Li et al. [7] introduced a temporal consistency constraint based on SRDCF, aiming to preserve the filter’s historical characteristics and thus prevent model degradation. Dai et al. [8] introduced an adaptive spatial regularization term that aims to dynamically adjust the regular spatial coefficients according to the changing target appearance. AutoTrack [15] works to uncover information hidden in the response map and adaptively change the regularity coefficients with local-global response variations. Augmented memory for correlation filters (AMCF) [39] penalized the non-target region filter coefficients by compressing the context, thus enhancing the model discrimination. Zhang et al. [44] combine saliency maps reflecting target appearance information and response maps reflecting tracking reliability into spatial regularization for dynamically penalizing non-target regions, thus optimizing the problem of fixed regularity failing to reflect changes in the target’s appearance. Based on STRCF [7], Jiang et al. [45] proposed to perceive variations in target appearance via saliency detection techniques, thus dynamically adjusting filter templates and spatial constraints to mitigate the risk of boundary effects and model degradation. 

### 2.3. DCF Tacking with Background Suppression

The issue of background interference has been plaguing the field of object tracking. Recently, the main research lines include feature-based and response-based suppression. Chen et al. [22] proposed a novel tracker that performs background filtering in the feature space using a background-suppressed (BS) function. Liu et al. [46] proposed Marked DCF (MDCF), which masks the background area with the help of a rectangle so that only the target region is retained. Mueller et al. [47] proposed a context-aware correlation filter (CACF), which incorporates context information of target patches to serve as negative samples, thus enhancing the discriminatory ability against background interference. Huang et al. [48] proposed a novel tracker with background suppression (BSCF), unlike the aforementioned tracker, which uses a mask matrix to mask the target region while only the background region is retained. The pure background contextual information is utilized as a training sample for the additional regularization term, allowing BSCF to suppress the background response and improve the model discrimination. Lin et al. [16] proposed response-based bidirectional incongruity error, allowing it to efficiently learn variations in appearance while gracefully suppressing background interference. Huang et al. [17] proposed aberrance repressed correlation filters (ARCF), which is to suppress response distortion due to background interference by minimizing the Euclidean distance between global response maps of consecutive frames. Zhang et al. [49] proposed the target-aware background-suppressed correlation filters (TABSCF), which generate a binary mask matrix by employing saliency detection algorithm to distinguish target region from the background. Furthermore, it employs the dual regression model to train the filter so as to suppress background response fluctuations. Zhang et al. [50] proposed a novel tracker with response-consistent and background-suppression, which uses the known response of the previous frame as a consistency reference to guide the construction of the filter, and introduces an attention mask matrix to enhance the perception of background information. Fu et al. [51] introduces the saliency detection method to construct the saliency perception regularity constraint of the target by perceiving the change of the appearance of the object, so as to achieve highlighting the appearance of the target while suppressing the irrelevant background noise.

## 3. Proposed Method

### 3.1. Short Review of the DCF Tracker

The traditional DCF optimizes the tracking model by solving a ridge regression problem so that the sum of squared error between the trained filter output and its corresponding label is minimized. It can be expressed as follows:

(1) $$\underset{h}{min}\sum_{i}^{n}{\left(f\left(x_{i}\right)-y_{i}\right)}^{2}+\lambda {\left\|h\right\|}_{2}^{2}$$

where $x_{i}$ denotes the n-dimensional feature maps of size *M* × *N* extracted from the input image by cyclic shift operation, $y_{i}$ denotes the regression label with a Gaussian shape. *λ* is the regularization parameter, which is used to prevent the classifier from overfitting. DCF treats the tracking task as a classification problem, so it usually introduces linear classifier $f\left(x_{i}\right)=h^{T}x_{i}$, h denotes the optimized filter coefficients.

Although the periodic hypothesis solves the problem of insufficient training samples, it also leads to sub-optimal filter performance due to the large number of unrealistic background samples. In addition, the tracker results are prone to drift in the presence of background interference. Two representative works focusing on these problems have been developed, namely the spatially regularized discriminative correlation filter (SRDCF) [6] and the aberrance repressed correlation filter (ARCF) [17].

SRDCF introduced a spatial regularization term to penalize the non-target region, making it possible to obtain more training samples in a larger search region without introducing too much background information. And the response map of SRDCF is more concentrated on the target region than traditional DCF, which results in more reliable tracking results. The objective function of SRDCF is as follows:

(2) $$\varepsilon \left(h\right)=\frac{1}{2}{\left\|y-\sum_{d=1}^{D}x^{d}*h^{d}\right\|}_{2}^{2}+\sum_{d=1}^{D}{\left\|w⊙h^{d}\right\|}_{2}^{2}$$

where *w* is a spatial constraint matrix with a negative Gaussian shape. Due to the introduction of an additional coefficient, which destroys the closed solution of traditional DCF. For it, SRDCF uses the Gauss-Seidel method to iteratively solve the objective function. This is the real reason for the low tracking speed of SRDCF, which is about 5 fps.

ARCF takes into account both the boundary effects and the response aberration. Firstly, it inherits the advantages of BACF by applying the binary mask matrix to crop out the image patches in the center of the search region to obtain more high-quality negative samples and effectively mitigate the influence of boundary effects. Meanwhile, it aims to suppress the response aberration by minimizing the Euclidean distance between the global response maps of the front and back frames, which improves the tracking performance in complex scenes. The objective function of ARCF is as follows:

(3) $$\begin{array}{c} E\left(h_{k}\right)=\frac{1}{2}{\left\|y-\sum_{d=1}^{D}B\;x_{k}^{d}*h_{k}^{d}\right\|}_{2}^{2}+\frac{\lambda }{2}\sum_{d=1}^{D}{\left\|h_{k}^{d}\right\|}_{2}^{2}\\ \;\;\;\;\;\;+\frac{\gamma }{2}{\left\|\sum_{d=1}^{D}\left(B\;x_{k-1}^{d}*h_{k-1}^{d}\right)\left[\psi_{p,q}\right]-\sum_{d=1}^{D}B\;x_{k}^{d}*h_{k}^{d}\right\|}_{2}^{2} \end{array}$$

where B is the binary mask matrix. ψ denotes the shift operator. q and p denote the maximum peak position difference between the front and back frame response maps, respectively. λ, γ denote the coefficient of each regular term. However, ARCF is implemented on the assumption that the response map of the previous frame is not contaminated. With the gradual accumulation of background interferences, the response map relying on the prior information constraints will become less reliable, and its suppression ability for anomalies will be limited.

### 3.2. The Proposed BSDCF Tracker

To address the issue of boundary effects and background interference, this work proposed a background-suppressed dual-regression correlation filter (BSDCF) for visual tracking to effectively obtain more stable and reliable tracking performance. Firstly, by constructing the filter penalty matrix *w* to concentrate the responses in the central region, thus effectively mitigating the background effects. Secondly, we introduced the background response suppression regularition to limit the variation rate of the global response, so as to achieve the purpose of highlighting the target and suppressing the background.The objective function of BSDCF is as follows:

(4) $$\begin{array}{c} \Theta \left(h_{o},h_{m}\right)=\sum_{n}^{\;}\left(\frac{1}{2}{\left\|y-\sum_{d=1}^{D}B\;x_{n}^{d}*h_{n}^{d}\right\|}_{2}^{2}+\frac{\lambda }{2}\sum_{d=1}^{D}{\left\|w⊙h_{n}^{d}\right\|}_{2}^{2}\right)\\ \;\;\;\;\;+\frac{\gamma }{2}{\left\|\sum_{d=1}^{D}B\;x_{o}^{d}*h_{o}^{d}-\sum_{d=1}^{D}B\;x_{m}^{d}*h_{m}^{d}\right\|}_{2}^{2},n\in \left(o,m\right) \end{array}$$

where $x_{o}$ and $x_{m}$ denote the global and target features, respectively, while $h_{o}$ and $h_{m}$ denote the trained global filter and target filter, respectively. The target features $x_{m}$ can be generated by the global features $x_{o}$ multiplying with background suppressed function. Several functions can be candidates for the background suppressed function to mask the background region, including the two-dimensional Gaussian function, the cosine function, or the simplest binary mask matrix.

**Figure 2:**
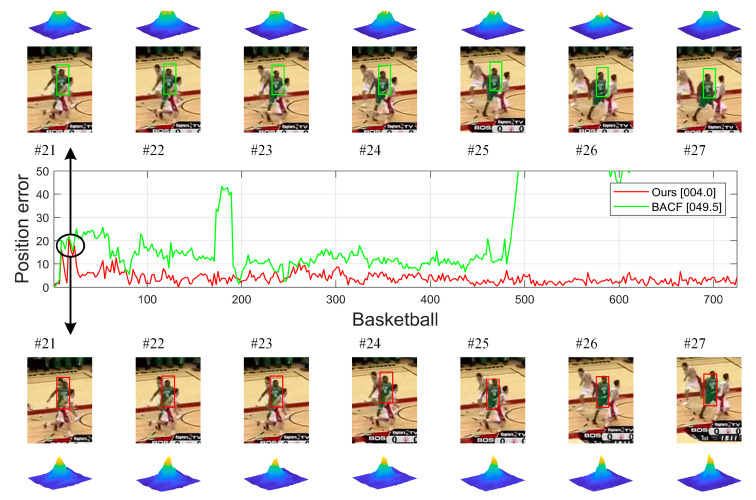
Comparison between the baseline BACF and the proposed BSDCF. The center figure shows their position error curves in the Basketball sequence.

When the target characteristics are relatively obvious in the search region, the filter will output the ideal response map with an approximately normal distribution. As shown in Figure 1, by cropping the target region out of the global image via a binary mask matrix, the resulting target response map has strong response values in the target region, while the response values in the background region tend to be zero. Therefore, exploiting the target response of the current frame to restrict the global response allows the model to reduce the accumulation of inter-frame errors while minimizing the background fluctuations in the global response. Moreover, since the target filter learns enough target features, the target response can also be used as an assistant to enhance the expression of the global response in the detection phase, resulting in larger response values for the target region. It can be seen from Figure 2 that the output response of the BACF tracker appears multi-peaked when the target is obscured, resulting in a skewed tracking result. While the proposed BSDCF can suppress the background response and obtain a prominent main peak, thus obtaining better tracking performance.

### 3.3. Optimization

The global filter $h_{o}$ and the target filter $h_{m}$ are solved iteratively by the alternating direction multiplier method (ADMM). In the process of solving $h_{o}$, it is assumed that $h_{m}$ is known, and the output response of the target filter is expressed as $R_{m}=\sum_{d=1}^{D}B\;x_{m}^{d}*h_{m}^{d}$. Thus, the objective function for solving $h_{o}$ is as follows:

(5) $$\Theta \left(h_{o}\right)=\frac{1}{2}{\left\|y-\sum_{d=1}^{D}B\;x_{o}^{d}*h_{o}^{d}\right\|}_{2}^{2}+\frac{\lambda }{2}\sum_{d=1}^{D}{\left\|w⊙h_{o}^{d}\right\|}_{2}^{2}+\frac{\gamma }{2}{\left\|\sum_{d=1}^{D}B\;x_{o}^{d}*h_{o}^{d}-R_{m}\right\|}_{2}^{2}$$

translated Equation (5) into frequency domain representation according to Parseval’s theorem:

(6) $$\hat{\Theta }\left(h_{o},{\hat{g}}_{o}\right)=\frac{1}{2T}{\left\|\hat{y}-\sum_{d=1}^{D}{\hat{x}}_{o}^{d}*{\hat{g}}_{o}^{d}\right\|}_{2}^{2}+\frac{\lambda }{2}\sum_{d=1}^{D}{\left\|w⊙h_{o}^{d}\right\|}_{2}^{2}+\frac{\gamma }{2}{\left\|\sum_{d=1}^{D}{\hat{x}}_{o}^{d}*{\hat{g}}_{o}^{d}-R_{m}\right\|}_{2}^{2}$$

where, ${\hat{g}}^{d}$ denotes an auxiliary variable, ${\hat{g}}^{d}=\sqrt{T}FB^{T}h^{d}$. The superscript ˆ denotes the discrete Fourier operator, and F denotes the Fourier transform matrix. Rewriting Equation (6) via the augmented Lagrange multiplier method (ALM) as:

(7) $$L\left(h_{o},{\hat{g}}_{o},\hat{\zeta }\right)=\hat{\Theta }\left(h_{o},{\hat{g}}_{o}\right)+\hat{\zeta }\left({\hat{g}}_{o}-\sqrt{T}PF^{T}h_{o}\right)+\frac{\mu }{2}{\left\|{\hat{g}}_{o}-\sqrt{T}PF^{T}h_{o}\right\|}_{2}^{2}$$

where μ denotes the penalty factor, and ζ denotes the Lagrange multiplier.

To obtain the closed-form solutions, Equation (7) will be split into the following three subproblems.

#### 3.3.1. Subproblem $h_{o}^{*}$

If ${\hat{g}}_{o}^{*}$ and $\hat{\zeta }$ are given, then the object function is the following:

(8) $$\varepsilon_{h_{o}^{*}}=\frac{\lambda }{2}{\left\|w⊙h_{o}\right\|}_{2}^{2}+\hat{\zeta }\left({\hat{g}}_{o}-\sqrt{T}PF^{T}h_{o}\right)+\frac{\mu }{2}{\left\|{\hat{g}}_{o}-\sqrt{T}PF^{T}h_{o}\right\|}_{2}^{2}$$

The solution of the above equation for the first-order derivative is as follows:

(9) $$\frac{\partial \varepsilon_{h_{o}^{*}}}{\partial h_{o}}=\lambda W^{T}Wh_{o}-\sqrt{T}PF^{T}\hat{\zeta }-\mu \sqrt{T}PF^{T}{\hat{g}}_{o}+T\mu$$

where W is the diagonal matrix form of w, i.e., W=diag(x).

(10) $$\begin{array}{c} h_{o}^{*}={\left(T\mu +\lambda_{1}W^{T}W\right)}^{-1}\left(\sqrt{T}PF^{T}\hat{\zeta }+\mu \sqrt{T}PF^{T}{\hat{g}}_{o}\right)\\ \;={\left(\mu I_{o}+\frac{\lambda_{1}w^{T}w}{T}\right)}^{-1}\left(\zeta +\mu g_{o}\right) \end{array}$$

where ζ and g can be obtained via the Fourier inverse operation on $\hat{\zeta }$ and ${\hat{g}}_{o}$.

(11) $$\left\{\begin{array}{c} \zeta =\frac{1}{\sqrt{T}}PF^{T}\hat{\zeta }\\ g_{o}=\frac{1}{\sqrt{T}}PF^{T}{\hat{g}}_{o} \end{array}\right.$$

#### 3.3.2. Subproblem ${\hat{g}}_{o}^{*}$

Since each element $x_{o}^{d}$ of the feature matrix is relatively independent, the subproblem ${\hat{g}}_{o}^{*}$ can be further split into *MN* subproblems:

(12) $$\begin{array}{c} {\hat{g}}_{o}^{*}(d)=\underset{g_{o}}{ar\;gmin}\{\frac{1}{2T}{\left\|\hat{y}\left(d\right)-{\hat{x}}_{o}^{T}\left(d\right){\hat{g}}_{o}\left(d\right)\right\|}_{2}^{2}+\frac{\gamma }{2}{\left\|{\hat{x}}_{o}^{T}\left(d\right){\hat{g}}_{o}\left(d\right)-R_{m}\right\|}_{2}^{2}\\ \;\;\;+{\hat{\zeta }}^{T}\left({\hat{g}}_{o}\left(d\right)-\sqrt{T}FP^{T}h_{o}\left(d\right)\right)+\frac{\mu }{2}{\left\|{\hat{g}}_{o}\left(d\right)-\sqrt{T}FP^{T}h_{o}\left(d\right)\right\|}_{2}^{2}\} \end{array}$$

The derivation yields:

(13) $$\begin{array}{c} {\hat{g}}_{o}^{*}\left(d\right)={\left[\left(\gamma +\frac{1}{T}\right){\hat{x}}_{o}\left(d\right){{\hat{x}}_{o}}^{T}\left(d\right)+\mu \right]}^{-1}\\ \;\;\;\;\;\;\;\;\;\cdot \left(\frac{1}{T}{\hat{x}}_{o}\left(d\right){\hat{y}}_{o}\left(d\right)+\gamma {\hat{x}}_{o}\left(d\right)R_{m}\left(d\right)-\hat{\zeta }+\mu {\hat{h}}_{o}\left(d\right)\right) \end{array}$$

Further, to simplify Equation (13) by the Sherman-Morrison formula:

(14) $${\left(uv^{T}+A\right)}^{-1}=A^{-1}-\frac{A^{-1}uv^{T}A^{-1}}{1+v^{T}A^{-1}u}$$

where u and v are two-column vectors and $uv^{T}$ is a rank one matrix. 

Finally, ${\hat{g}}_{o}^{*}\left(d\right)$ is derived as:

(15) $$\begin{array}{c} {\hat{g}}_{o}^{*}\left(d\right)=\frac{1}{\mu }\left[\frac{1}{T}{\hat{x}}_{o}{\hat{y}}_{o}+\gamma {\hat{x}}_{o}R_{m}-\hat{\zeta }+\mu {\hat{h}}_{o}\right.\\ \;\;\;\;\;\;-\frac{{\hat{x}}_{c}}{B}\left.\left(\frac{1}{T}{\hat{S}}_{xx}{\hat{y}}_{o}+\gamma {\hat{S}}_{xx}R_{m}-{\hat{S}}_{x\zeta }+\mu {\hat{S}}_{xh}\right)\right] \end{array}$$

here set ${\hat{S}}_{xx}={\hat{x}}^{T}\hat{x}$, ${\hat{S}}_{x\zeta }={\hat{x}}^{T}\hat{\zeta }$, A=γ+1/T, $B=\mu /A+{\hat{S}}_{xx}$, ${\hat{S}}_{xh}={\hat{x}}^{T}\hat{h}$.

#### 3.3.3. Lagrange Multiplier $\hat{\zeta }$

The Lagrange multiplier $\hat{\zeta }$ is determined by the following:

(16) $${\hat{\zeta }}_{i+1}={\hat{\zeta }}_{i}+\varphi \left({\hat{g}}_{i+1}^{*}-{\hat{h}}_{i+1}^{*}\right)$$

where the subscript i is the number of iterations. 

To speed up the convergence of the model optimization, the multiplicative factor *φ* is updated in the following way:

(17) $$\varphi^{i+1}=\min\left(\varphi_{max},\beta \varphi^{i}\right)$$

#### 3.3.4. Algorithm Complexity Analysis

Each element of the feature matix is relatively independent in the ADMM iteration process, so only the D*MN subproblems must be solved, where D represents the number of feature channels. The subproblem ${\hat{g}}_{o}^{*}$ requires FFT and IFFT transforms in each iteration, so the computational complexity is *O*(D*MNlog(MN)). The computational complexity of the subproblem $h_{o}^{*}$ is *O*(D*MN). The update process of the Lagrange multiplier $\hat{\zeta }$ is linearly computed, thus, the computational complexity is *O*(1). Since the spatial regularity coefficient *w* is a pre-set constant term, there is no additional computational demand. In summary, assume that the number of iterations is set to *L* and the overall complexity of the proposed BSDCF is *O*(LD*MNlog(MN)).

### 3.4. Object Detection and Model Update

To strengthen the response intensity in the target region, the final output response $R_{c}$ is obtained by the weighted fusion of the two response maps:

(18) $$R_{c}=F^{-1}\left(\sum_{d=1}^{D}{\hat{h}}_{o-1}^{d*}⊙{\hat{x}}_{o}^{d}+\tau \cdot {\hat{h}}_{m-1}^{d*}⊙{\hat{x}}_{m}^{d}\right)$$

where $F^{-1}$ denotes the Fourier inverse operation, and *τ* is the fusion coefficient. The maximum point of the final response map will be the object position.

Like the literature [7,12,28], we update the global and target appearance models with a pre-set learning rate, expecting the trained filter to learn part of the target’s history information, thus improving the filter’s robustness in complex tracking scenarios.

(19) $${\hat{X}}_{n}^{model,t}=\left(1-\eta \right){{\hat{X}}_{n}}^{model,t-1}+\eta {\hat{x}}_{n},n\in \left(o,m\right)$$

where $X_{n}$ denotes the updated appearance model, t and t−1 denote the *t*-th frame and (*t*−1)-th frame, respectively, and η denotes the learning rate of the appearance model.

The overall workflow of the proposed BSDCF is presented in Algorithm 1.
**Algorithm 1.** Background-suppressed dual-regression correlation filters (BSDCF).**Input**: The first frame target state (including target position $T_{1}$ and scale information $S_{1}$); **Output**: The *t*-th frame target position $T_{t}$ and scale information $S_{t}$;**1**Initialize model hyperparameters.**2****for***t* = 1: end **do****3** **Training**
**4** Determining the search region and extracting global features $x_{o}.$
**5** Calculate target mask matrix and obtain target features $x_{m}.$
**6** **for** *Iter* = 1: *L* **do****7**  Optimize the filter model $h_{m}$ via Equations (10) and (15)–(17).**8**  Obtained target response $R_{m}$ via the target filter $h_{m}$ correlated with the target appearance model $X_{m}$.**9**  Optimize the filter model $h_{o}$ via Equations (10) and (15)–(17).**10**  Obtained target response $R_{o}$ via the target filter $h_{o}$ correlated with the target appearance model $X_{o}$.**11** 
**end for**
**12**  **Detecting**
**13** Cropping the search region at N different scales, centered on the target position $T_{t-1}$ obtained at frame (*t*−1).**14** Crop multi-scale search regions centered at $P_{t-1}$ with *S* scales based on the bounding box at frame *t*.**15** Extract multi-scale global feature maps $x_{o}^{r}$.**16** Calculate target mask matrix and obtain multi-scale target features $x_{m}^{r}.$
**17** Use Equation (18) to final response map $R_{r},(r=1,2,\ldots ,S)$.**18** Prediction of the target location $T_{t}$ and best scale $S_{t}$ based on the maximum peak of the response map.**19** Use Equation (19) to update the appearance model.**20****end for**

## 4. Experiments

In this section, the tracking performance of the proposed BSDCF is evaluated using the one-pass evaluation (OPE) criterion on three widely used benchmark datasets, which are OTB100 [52], Temple-Color 128 [53], and UAVDT [54]. The SOTA trackers that participated in the comparison include STRCF [7], ECO_HC [28], LADCF_HC [14], BACF [10], ARCF [17], AutoTrack [15], Staple [32], BiCF [16], SRDCF [6], fDSST [35], AMCF [39], SAMF [36], and KCF [12]. The evaluation metrics include success rate and distance accuracy (DP), as well as the tracking speed measured in frames per second (FPS).

### 4.1. Implementation Details

The experiments involved were conducted in the same run-time environment with a CPU of Intel i7-7700 3.60 GHz, GPU of NVIDIA GT730, memory of 16.00 GB, operating system of 64-bit Window 11, and test software of MATLAB R2017b. For the proposed BSDCF, the background response suppression regularization coefficient γ was set to 0.92, the fusion coefficient τ was set to 0.01, the iteration number of ADMM was set to 2, and the appearance model learning rate η was 0.019. The proposed BSDCF used HOG color names features for position estimation and extracted 5 scale size HOG features for scale estimation. The rest of the parameters were consistent with the baseline BACF tracker’s settings.

### 4.2. Overall Performance

**Figure 3:**
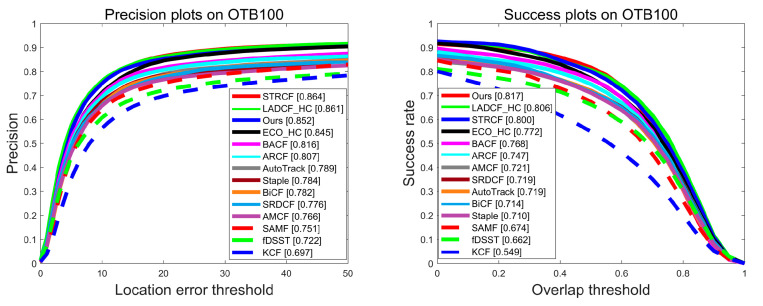
Evaluation results in terms of precision and success plots on OTB100 benchmark.

**Evaluation on OTB100 Benchmark**. The OTB100 benchmark consists of 100 sets of fully labeled image sequences, totaling over 58,000 images. While it further classifies 11 kinds of challenging attributes for image sequences, including Scale Variation (SV), Occlusion (OCC), Illumination Variation (IV), Low Resolution (LR), Deformation (DEF), Out-of-Plane Rotation (OPR), Fast Motion (FM), Motion Blur (MB), In-Plane Rotation (IPR), Out-of-View (OV), and Background Clutters (BC). Each set of sequences contains one or more challenge attributes. The overall evaluation results on the OTB100 are shown in Figure 3. It can be seen that the proposed BSDCF ranks third (85.2%) and best (81.7%) in terms of precision and success rate, respectively. Compared with the baseline BACF, the performance in terms of precision and success rate is improved by 3.6% and 4.9%, respectively. The tracking performance of ARCF, which improves the BACF by utilizing the before-and-after frame response constraint strategy, reduces the tracking performance on the OTB100. Especially, the proposed BSDCF is 4.5% and 7.0% better than ARCF in terms of precision and success rate, respectively.

**Figure 4:**
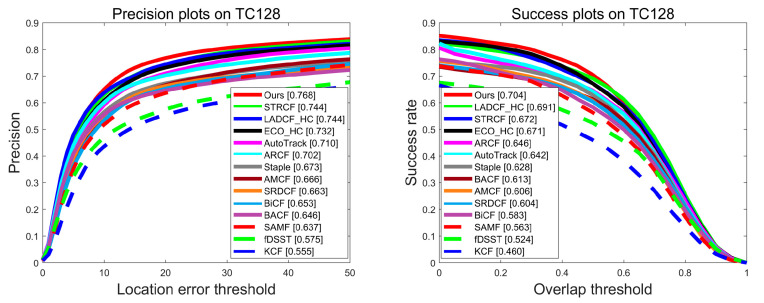
Evaluation results in terms of precision and success plots on the TC128 benchmark.

**Evaluation on TC128 Benchmark**. The TC128 (temple color) benchmark evaluates the effect of different color spaces on the tracker, and it collects 128 sets of full-color image sequences about 27 object classes. The overall evaluation results on the TC128 benchmark are shown in Figure 4. It can be seen that the proposed BSDCF both ranks first in terms of precision and success rate (76.8% and 70.4%). Compared with the baseline BACF, the proposed improves by 12.2% in terms of precision and 9.1% in terms of success rate. Compared with ARCF, the proposed is 6.6% and 5.8% higher in terms of precision and success rate, respectively.

**Figure 5:**
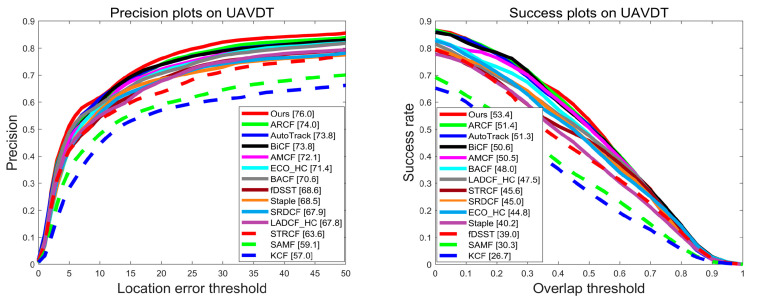
Evaluation results in terms of precision and success plots on UAVDT benchmark.

**Evaluation on UAVDT Benchmark**. The UAVDT (UAV detection and tracking) benchmark collects 50 sets of vehicle image sequences captured by UAVs, with a total frame count of over 80,000. Compared to the OTB100, the complex weather conditions (e.g., rain, night), the small size, large scale variation, and similar objects poses a greater challenge to trackers. The overall evaluation results on the UAVDT are shown in Figure 5. It can be seen that the proposed BSDCF both ranks first in terms of precision and success rate (76.0% and 53.4%). Compared with the baseline BACF, BSDCF improves by 5.4% and 5.4% in terms of precision and success rate, respectively. Due to the significant difference between the image sequences from the UAV viewpoint and those acquired by ordinary cameras, ARCF, AutoTrack, BiCF, and AMCF, which are improved for the characteristics of the UAV datasets, have significantly higher precision and success rate rankings, while LADCF_HC and STRCF, which perform well in the conventional dataset, have significantly lower rankings. Nevertheless, the proposed BSDCF still maintains a good tracking performance. Compared with the second-ranked ARCF, BSDCF is 2.0% and 2.0% higher in terms of precision and success rate, respectively.

**Overall evaluation**. Table 1 lists the average accuracy scores and tracking speed of the top eight tracking trackers based on the OTB100, TC128, and UAVDT benchmarks. It can be seen that the proposed BSDCF ranks first in terms of average precision and average success rate, with 79.3% and 68.5%, respectively. Compared with the baseline BACF, the proposed improves the precision by 7.1% and the success rate by 6.5%. Compared with the ARCF, the proposed is 4.4% and 5.0% better regarding distance accuracy and tracking success rate, respectively.

### 4.3. Attribute Evaluation

To further validate the performance of the proposed BSDCF under different tracking scenarios, in this section, the top eight trackers in terms of tracking performance are selected to be experimental on 11 challenge attributes of the OTB100 benchmark for attribute evaluation. The involved trackers include STRCF, ECO_HC, BACF, LADCF_HC, SRDCF, AutoTrack, ARCF, and the proposed BSDCF. Table 2 and Table 3 show the evaluation results on the precision and success rate of the OTB100 challenge attributes, respectively. It can be seen that the proposed BSDCF performs well in different tracking scenarios. Especially, the precision and success rate scores are ranked first in the in-plane rotation (IPR), illumination change (IV), background clutter (BC), and fast motion (FM) tracking scenarios. Compared with the baseline BACF, the precision and success rate in different scenarios are improved by 3% to 5%, proving that introducing spatial regularization and background response suppression regularization is effective.

### 4.4. Qualitative Evaluation

**Figure 6:**
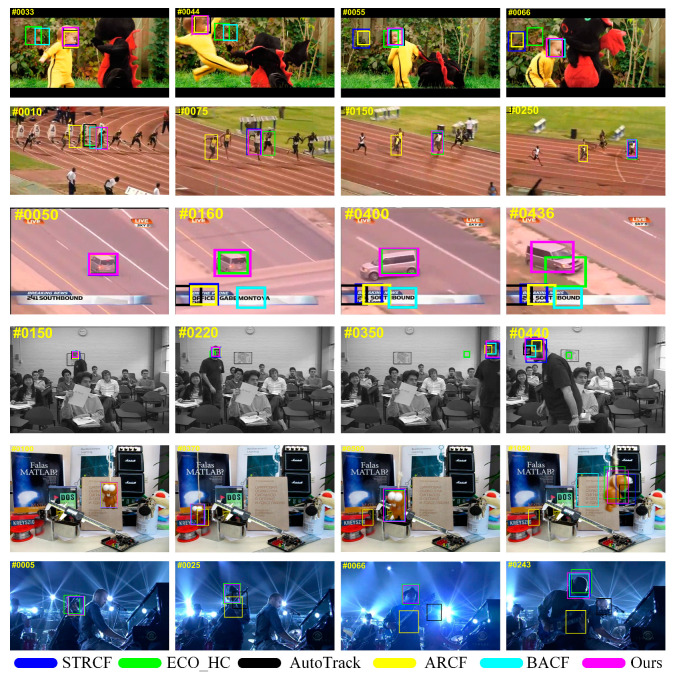
Qualitative evaluation of the proposed BSDCF with top-5 trackers in 6 challenging video sequences, including DragonBaby, Bolt2, Carchasing_ce1, Freeman3, Lemming, and Shanking (from top to bottom).

To demonstrate the tracking performance of the proposed BSDCF more intuitively, this section provides the qualitative evaluation by comparing with STRCF, ECO_HC, AutoTrack, ARCF, and BACF on six sets of challenging sequences, which include DragonBaby, Bolt2, Carchasing_ce1, Freeman3, Lemming, Shanking. The running results of some image frames are shown in Figure 6. Experiments demonstrate that the proposed can perform well in different tracking scenarios, especially in challenging scenarios such as fast motion, scale changes, illumination changes, and occlusion.

(1) Fast motion: Representative sequences of this attribute include DragonBaby, Shanking, and Lemming. Fast motion challenges are often accompanied by target blurring, deformation, etc., and are extremely susceptible to tracking failure, such as frame 500 in the Lemming sequence and frames 66 and 243 in shaking. In the DragonBaby sequence, the target moves its body quickly and changes position drastically in the view, which both pose a great challenge. However, the proposed BSDCF is still able to track accurately.
(2) Scale change: The Carchasing_ce1 and Freeman3 both belong to this attribute. Due to the Dual model and spatial regularization to reinforced target regions, the proposed can quickly adapt to the target’s scale change. Similar to the 380th and 436th frames in the Carchasing_ce1 sequence, as well as the 220th and 440th frames in the Freeman3 sequence, the proposed BSDCF can forecast the target state well when the target itself makes a large turn or makes a U-turn behavior.
(3) Occlusion: The DragonBaby, Carchasing_ce1, and Lemming all belong to this attribute. By suppressing the response aberration through the target response and thus highlighting the target region, the occlusion challenge is effectively addressed. For example, the proposed BSDCF can locate the target at 370 frames in the Lemming sequence, 160 frames in the Carchasing_ce1 sequence, and 55 frames in the DragonBaby sequence.
(4) Others: The proposed is relatively well adapted to illumination changes and background clutter challenges. In a representative shaking sequence, the target in a backlit environment is accompanied by a bright light that prevents the target’s appearance from being visible in the view. In the Bolt2 sequence, the interference of similar objects makes the interested target less prominent. Despite these challenges, the proposed BSDCF can still achieve relatively satisfactory tracking performance.

### 4.5. Effectiveness Evaluation

#### 4.5.1. Key Parameters Analysis

**Figure 7:**
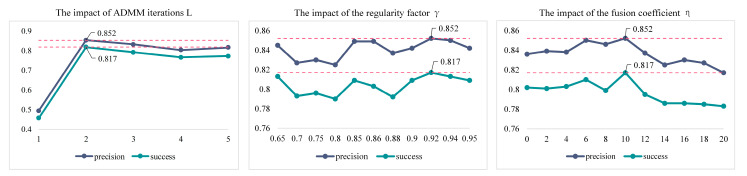
The precision and success rate scores of different key parameters in OTB100.

To verify the rationality of the key parameter settings, this section will do a validity analysis of the key parameters on the OTB100 benchmark. The overall evaluation result is shown in Figure 7. 

To obtain the best convergence experimental results, this work is conducted on the OTB100 benchmark for five rounds. It can be seen from Figure 7 that the proposed BSDCF requires only two rounds of ADMM iterations to obtain the optimal performance.

The background response suppression regularization coefficient γ is firstly taken in larger steps (0.5) in the interval [0.65, 0.95]. It is then carefully divided. Since the tracking accuracy is relatively large in the interval [0.85, 0.95], the experiment is repeated with smaller step sizes (0.2). From the experimental results, it can be seen that the tracking accuracy fluctuates slightly with the change of γ, and the precision and success rate reach the maximum when γ equals 0.92. 

Since the target filter is trained using only target features, the lack of background information learning leads to its limited discriminative ability. If the fusion coefficient η is set too large, it tends to be counter-productive. Therefore, the target response is only used to enhance the global response. As η rises from zero value, the accuracy curve also grows gradually. However, the accuracy curve drops sharply when η is greater than 0.01.

#### 4.5.2. Ablation Study

To verify the effectiveness of different modules for the proposed BSDCF, this section will split different modules for ablation experiments. BACF serves as the “Baseline”, “Baseline-W” indicates the addition of the spatial regularization module to “Baseline”, and “Baseline-B” indicates the addition of the background response suppression regularization module. “Ours” represents the proposed complete tracking framework, which contains all modules.

The overall result of the ablation experiments is shown in Table 4. As can be seen from the table, Baseline achieves 72.2% and 63.8% in average precision and success rate, respectively. Benefiting from the spatial regularity term, Baseline-W achieves 5.0% and 3.0% higher average precision and success rate, respectively, compared with Baseline. Meanwhile, Baseline-B also outperforms the benchmark by 5.7% and 3.5% in terms of average precision and success rate, respectively, indicating that suppression of background by target response is effective for the overall tracker performance improvement. With the introduction of all key modules, the average precision and success rate of “Ours” reach the optimum. All the above experimental results show the effectiveness of each module.

## 5. Conclusions

In this work, we propose a novel background-suppressed dual-regression correlation filter (BSDCF) for visual tracking, which adopts the overall strategy of exploiting the target response to suppress the background response for addressing the aberration of the filter response due to background interference. Especially, the global filter and the target filter are trained separately by the dual regression model. The target response is “prominent in the target region and flat in the background region” to limit the fluctuation of the global response caused by background interference. Meanwhile, the spatial regularization term is introduced to further highlight the target region. Finally, the tracking performance is verified on three widely used benchmarks (OTB100, TC128, UAVDT). The experimental results demonstrate that the proposed BSDCF can perform excellent tracking performance in different tracking scenarios. Due to the strong expressive power of deep features, future work will try to incorporate deep learning into the correlation filters to obtain better tracking performance.

## Figures and Tables

**Table 1:** Average accuracy and tracking speed performance of the top-8 trackers on the OTB100, TC128, and UAVDT benchmark.

Trackers	STRCF	ECO_HC	LADCF_HC	BACF	ARCF	AutoTrack	SRDCF	Ours
Precision	74.8	76.3	76.1	72.2	74.9	74.5	70.6	**79.3**
Success rate	64.2	63.0	65.7	62.0	63.5	62.4	59.1	**68.5**
FPS	25	**51**	20	39	17	47	7	22

The best results are shown in **bold**.

**Table 2:** Success rate performance of the top-8 trackers on the OTB100 benchmark.

	STRCF	ECO_HC	BACF	ARCF	SRDCF	AutoTrack	LADCF_HC	Ours
LR	69.7	73.0	69.5	67.9	69.0	71.4	69.2	63.3
IPR	74.5	68.5	71.6	68.3	64.4	67.1	74.1	76.1
OPR	76.8	72.3	70.9	68.3	65.2	67.3	77.8	77.8
IV	78.3	76.1	78.4	75.6	73.7	75.1	79.1	83.8
OCC	75.4	74.9	69.7	68.7	67.9	67.3	79.2	76.5
DEF	73.7	74.0	69.7	71.6	66.5	68.7	72.2	76.7
BC	79.6	76.2	75.9	74.2	67.8	70.9	79.7	86.5
SV	76.3	71.7	70.2	67.7	65.7	64.3	76.8	75.6
MB	79.4	75.3	73.5	74.2	71.1	70.0	79.3	79.9
FM	75.7	74.0	75.6	73.4	70.5	69.1	74.4	78.3
OV	69.3	65.7	68.9	62.5	55.3	63.6	71.5	75.9

The top three results are marked with “ ”, “ ”and “ ” respectively.

**Table 3:** Precision performance of the top-8 trackers on the OTB100 benchmark.

	STRCF	ECO_HC	BACF	ARCF	SRDCF	AutoTrack	LADCF_HC	Ours
LR	71.1	77.3	71.0	72.6	69.8	79.7	70.1	64.5
IPR	80.9	78.2	78.6	78.1	70.9	78.6	80.8	82.2
OPR	85.0	81.1	77.9	76.9	72.7	73.2	83.8	83.2
IV	84.1	79.8	80.8	76.6	76.7	79.2	80.8	85.4
OCC	81.4	81.0	73.5	74.0	71.1	70.4	83.0	78.8
DEF	84.4	82.3	76.9	76.9	72.6	70.1	81.2	81.6
BC	86.6	81.3	79.4	74.9	72.1	79.2	83.4	89.9
SV	84.2	80.8	77.1	77.1	73.2	74.3	83.6	81.0
MB	82.6	78.0	74.1	75.7	73.3	75.3	80.7	81.8
FM	80.2	79.2	78.7	76.8	75.1	74.8	79.0	81.3
OV	76.6	73.7	74.8	67.1	58.2	70.9	81.5	80.5

The top three results are marked with ” ”, “ ”and “ ” respectively.

**Table 4:** Overall evaluation performance of ablation experiments on the OTB100, TC128, and UAVDT benchmarks.

Trackers	OTB100		TC128		UAVDT		Average	
	Precision	Success	Precision	Success	Precision	Success	Precision	Success
Baseline	81.6	76.8	64.6	61.3	70.6	53.4	72.2	63.8
Baseline-W	83.1	79.1	75.8	69.7	72.7	51.7	77.2	66.8
Baseline-B	84.7	80.6	75.4	**70.7**	73.6	50.8	77.9	67.3
Ours	**85.2**	**81.7**	**76.8**	70.4	**76.0**	**53.4**	**79.3**	**68.5**

The best results are shown in **bold**.

## Data Availability

Not applicable.
